# Supplementary material for: A quantum radio frequency signal analyzer based on nitrogen vacancy centers in diamond
Source: Commun Eng. 2022 Jul 27;1:19. doi: 10.1038/s44172-022-00017-4 (PMC10955911; doi:10.1038/s44172-022-00017-4)
Supplement: Supplementary file 1 — Supplementary Information [file 44172_2022_17_MOESM1_ESM.pdf]

A quantum radio frequency signal analyzer based on nitrogen vacancy centers in diamond: Supplementary information

Simone Magaletti[1], Ludovic Mayer[1], Jean-François Roch[2], and Thierry Debuisschert[1,*]

[1]Thales Research and Technology, 1 Avenue Augustin Fresnel, Palaiseau Cedex, 91767, France
[2]Université Paris-Saclay, CNRS, ENS Paris-Saclay, CentraleSupelec, LuMIn, Gif-sur-Yvette, 91190, France
[*]thierry.debuisschert@thalesgroup.com

1 Supplementary Note 1. The source of the static magnetic field

As reported in the main text, we are interested in a room temperature magnetic field source which is able to generate both strong magnetic field and strong magnetic field gradient. The simplest solution that fulfils these requirements is a single neodymium magnet, which is the strongest type of commercial permanent magnets, with a remanent magnetization (B_r) of the order of 1.3 T. In our set-up we used a 6.35 mm radius spherical magnet, whose magnetic field along the magnetization axis is given by the formula:

$$B(d) = \frac{2}{3}\frac{B_r \cdot R^3}{(R+d)^3} \tag{1}$$

where R is the radius of the magnet and d is the distance to the magnet surface. The magnetic field gradient and the Zeeman shift of the NV centers resonance lines are reported on fig. 3a of the main text. With our choice, the strongest magnetic field felt by the NV centers is of the order of 800 mT, equivalent to a resonance frequency for the $|0\rangle \rightarrow |+1\rangle$ transition of 27 GHz, when the diamond is the closest possible to the magnet. In order to measure the CW-ODMR of the NV centers close to the magnet surface, the RF antenna is displaced with respect to the position used in the main text (Supplementary figure 1a) and the NV centers are excited by the RF field generated outside the current loop (Supplementary figure 1b). The ODMR spectra of the NV centers embedded in the diamond area defined by the antenna and the diamond surface at the contact with the magnet are reported in Supplementary figure 1c. The measurement demonstrates that the NV centers electron spin resonance can be detected up to 27 GHz using a neodymium magnet at room temperature. However, the configuration described in Supplementary figure 1b is disadvantageous for sensing because the amplitude of the RF field decreases fast outside the antenna. For this reason, in the main text we used the configuration described in Supplementary figure 1a.
The last point we want to discuss concerns the temperature dependence of the remanent magnetization B_r, which, for neodymium magnets, is given by [1]:

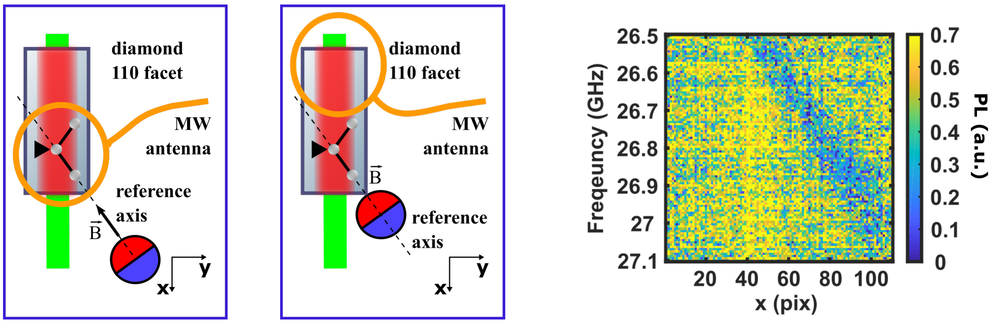

(a) (b) (c)

Supplementary figure 1: Maximum frequency detectable using our neodymium magnet. (a) Experimental configuration to perform the spectral analysis as it is described in the main text. The diamond area useful for sensing is the one inside the loop antenna. The PL (red color) results from the excitation of the laser (green color). (b) Experimental set-up to measure the strongest Zeeman shift possible with our neodymium magnet. The antenna is displaced in order to be able to move the magnet at the contact of the diamond and collect the PL emitted by the NV centers in proximity of the magnet surface. (c) ODMR spectra obtained employing the experimental configuration in (b).

$$\frac{dB_r}{dT} = -10^{-3} \cdot B_{\mathrm{r}} \tag{2}$$

namely the remanent magnetization decreases of -0.1% per degree. Therefore, temperature fluctuations induce magnetic field fluctuations. While the temporal resolution is small compared to temperature fluctuation characteristic times (tens of minutes), the longer calibration procedure can be affected by this effect. The main consequence is a broadening of the ODMR linewidth, which influences the frequency resolution.

2 Supplementary Note 2. The alignment procedure

The alignment procedure consists in aligning the magnetic field generated by the magnet with one of the NV center family. It is necessary in order to avoid the spin states mixing which changes the optical properties of the NV center, in particular reducing the emitted PL and the ODMR contrast [2]. Moreover, a good alignment induces a linear Zeeman shift of the energy levels which results in higher resonance frequencies and therefore a larger frequency range for the RF spectral detection. The spin states mixing can be understood looking at the Hamiltonian of the NV center interacting with a static magnetic field ($\vec{\mathbf{B}}$)

$$H = h(DS_z^2 + \gamma B_z S_z + \gamma(B_x S_x + B_y S_y)) \tag{3}$$

D is the zero field splitting (D = 2.87 GHz for the NV center ground state and D = 1.42 GHz for the NV center excited state); γ = 28 GHz$\cdot$T^{-1} is the NV center gyromagnetic ratio; $\vec{\mathbf{S}} = (S_x, S_y, S_z)$ is the spin operator; z is the direction of the NV center axis, namely the intrinsic spin quantization axis, and x and y are arbitrary chosen in a plane perpendicular to z. The first two terms of eq. (3) constitute the diagonal part of the Hamiltonian; the last two terms of eq. (3), different from zero when the magnetic field is not aligned with the NV center axis, are the

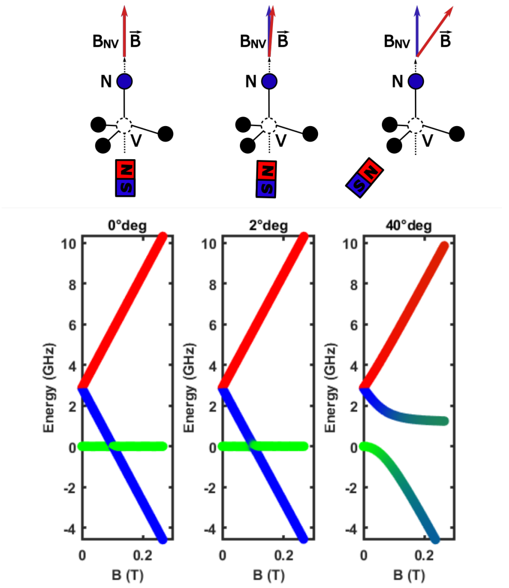

(a)

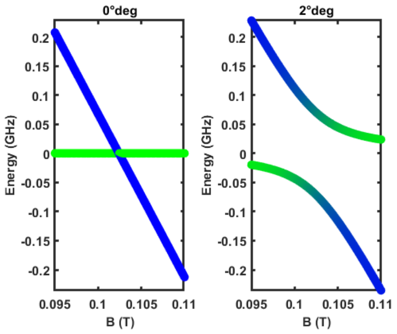

(b)

Supplementary figure 2: Hamiltonian eigenvalues. (a) NV center energy levels under a static magnetic field tilted of an angle θ (from left to right $\theta = 0°$; $\theta = 2°$; $\theta = 40°$). The color code is defined according to eq. (4) (b) Ground state level crossing (left) and ground state level anticrossing (right)

non-diagonal terms of the Hamiltonian. The eigenvalue of eq. (3) are plotted in Supplementary figure 2a for three different cases: a magnetic field perfectly aligned with the NV center axis ($B_x = B_y = 0$); a magnetic field slightly misaligned (2°) with respect to the NV axis; a magnetic field strongly misaligned (40°) with respect to the NV axis. The colour of each data point is expressed in the RGB scale by defining the components of the RGB three-dimensional vector as:

$$R = |\langle +1|\psi\rangle|^2; G = |\langle 0|\psi\rangle|^2; B = |\langle -1|\psi\rangle|^2 \qquad (4)$$

where $|\psi\rangle$ is the state vector of the system and $|0, \pm 1\rangle$ are the $[S_z, S^2]$ eigenstates.
Using this colour scale, the plot contains also the information about the eigenvectors of the system. In fact, a red point means that the system eigenstate associated to that eigenvalue is $|+1\rangle$, a green point means that the system eigenstate associated to that eigenvalue is $|0\rangle$ and a blue point means that the system eigenstate associated to that eigenvalue is $|-1\rangle$.
When the static magnetic field is perfectly aligned with the NV center, the eigenvector of the system are $|0, \pm 1\rangle$ and the Zeeman shift is linear with the amplitude of the static magnetic field. For a magnetic field $B = D/\gamma$ a level crossing is expected between the $|0\rangle$ and the $|-1\rangle$ states. In Supplementary figure 2a the eigenvalues are evaluated for the NV center ground state ($D = 2.87$ GHz) and therefore the ground state level crossing occurs at $B = 102$ mT. A similar behaviour is observed at the excited state, where the zero field splitting is $D_{\text{ES}} = 1.42$ GHz and the level crossing occurs at $B = 51$ mT. Both at the ground state and at the excited state, the level crossing is avoided in case of a misaligned static magnetic field. The ground state level anticrossing (GSLAC) is shown on Supplementary figure 2b and it is caused by the non diagonal terms of eq. (3). The mixing of the spin sublevels (as shown by the colour scale of the data points) induces a drop of PL and contrast in the ODMR spectrum. Moreover, the level anti

crossing induces an energy gap between the states whose crossing is avoided. The amplitude of this gap depends on the amplitude of the non diagonal terms of the NV center Hamiltonian with respect to the diagonal ones. Both the drop of contrast and the presence of an energy gap whose amplitude depends on the degree of misalignment can be exploited to align the static magnetic field with respect to the NV center axis. In proximity of the GSLAC the alignment is performed minimizing the energy gap, namely the smallest ODMR frequency detectable, as shown in Supplementary figure 3c and Supplementary figure 3d. It is important to remark that, due to the non-diagonal terms also induced by the hyperfine coupling between the NV center electron spin and the nitrogen nuclear spin, even in the case of a perfect alignment, the level crossing is still avoided [3]. However, in this case, the energy gap is rather small (some MHz in our case Supplementary figure 3d) and it finally corresponds to the lower limit of the MW frequency range we are able to detect using NV centers for RF spectral analysis. In proximity of the ESLAC, since the level anticrossing occurs at the excited state and we are probing the ground state, we are not able to directly observe the energy gap using the ODMR technique. However we can perform the alignment through an iterative procedure with the aim of maximizing the ODMR contrast of the ground state spectrum around 1.45 GHz (Supplementary figure 3c, and Supplementary figure 3d), which is the resonance frequency of the $|0\rangle \rightarrow |-1\rangle$ transition for a magnetic field of 51 mT.
A third possible alignment procedure is based on geometrical considerations. As explained in the main text, the NV center axis can be oriented along four different diamond crystallographic axes (fig. 1 of the main text). When the magnetic field is aligned along one of these axis, according to the tetrahedral structure of the diamond lattice, the magnetic field is tilted of the same angle with respect to the other three crystallographic axis. Therefore, according to eq. (3), the three non-aligned NV families are characterized by the same eigenvalues and the same ODMR spectrum. From an experimental point of view, the alignment procedure consists in tuning the magnet position until the ODMR spectra of the three non-aligned families are superimposed (Supplementary figure 4). This procedure works well for low static magnetic field, because in this case the three non-aligned families undergo a low transverse magnetic field that still enables the optical detection of their spin resonances. Under stronger magnetic fields, the transverse component applied to the three non-aligned families makes impossible the optical detection of their spin resonances.

3 Supplementary Note 3. Contrast improvement by means of a polarizer in the PL detection chain

The NV center radiative emission process is governed by two electric dipoles laying in the plane perpendicular to the NV center axis [5, 6]. Each dipole emits an electromagnetic field whose expression, in the far field regime [7], is:

$$\vec{\mathbf{E}}(\vec{\mathbf{r}}) = \frac{E_0}{r} e^{-i\omega t + ikr} (\vec{\mathbf{n}} \times \vec{\mathbf{p}}) \times \vec{\mathbf{n}} \tag{5}$$

where $\vec{\mathbf{n}}$ is a unit vector along the vector position direction $\vec{\mathbf{r}}$, $\vec{\mathbf{p}}$ is a unit vector along the electric dipole moment direction and E_0, k and ω are respectively the amplitude, the wave vector and the angular frequency of the electric field. Two different cases are considered: $\vec{\mathbf{n}} \perp \vec{\mathbf{p}}$ and $\vec{\mathbf{n}} \parallel \vec{\mathbf{p}}$. In the first case, that is when we are looking in a direction perpendicular to the dipole axis, the amplitude of the electric field is maximal ($|(\vec{\mathbf{n}} \times \vec{\mathbf{p}}) \times \vec{\mathbf{n}}| = 1$) and the electric field is polarized along the dipole axis ($(\vec{\mathbf{n}} \times \vec{\mathbf{p}}) \times \vec{\mathbf{n}} = \vec{\mathbf{p}}$). In the second case the electric field is null. At room temperature, the two NV center dipoles are coupled and both of them can emit independently

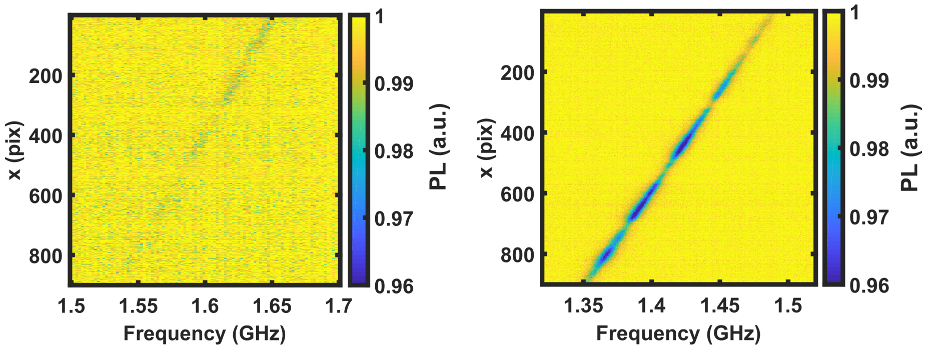

(a) *ESLAC misaligned* (b) *ESLAC aligned*

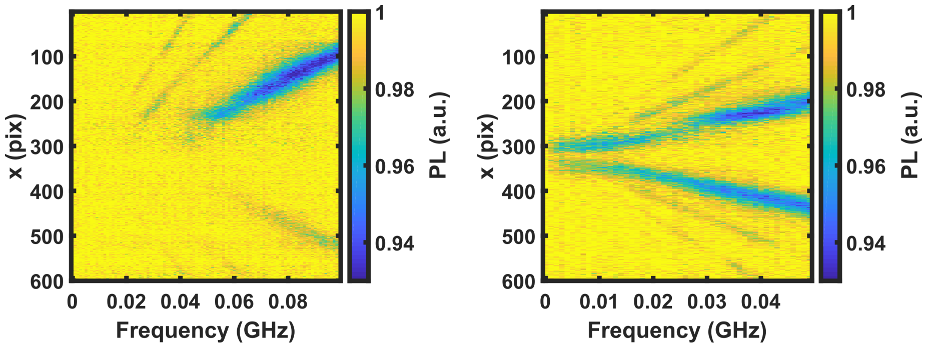

(c) *GSLAC misaligned* (d) *GSLAC aligned*

Supplementary figure 3: Alignment procedure at the level anti-crossing. (a-b) Ground state $|0\rangle \rightarrow |-1\rangle$ ODMR spectrum in proximity of the ESLAC in case of misaligned (a) and aligned (b) static magnetic field. At the ESLAC the ground state $|0\rangle \rightarrow |-1\rangle$ transition is resonant at 1.45 GHz. In case of a misaligned magnetic field the loss of contrast makes impossible the NV center ODMR of the $|0\rangle \rightarrow |-1\rangle$ transition also for resonance frequencies hundreds of MHz lower or higher than 1.45 GHz. The sensitivity to the alignment at the ESLAC is so high ([4]) that, in (b), the resonance line is very well visible at 1.42 GHz but not at 1.5 GHz. That happens because the static magnetic field is better aligned to the NV centers in the lower part of the image ($x = 900$) than to the ones at top of the image. (c-d) Ground state $|0\rangle \rightarrow |-1\rangle$ ODMR spectrum in proximity of the GSLAC respectively when the magnet is misaligned (c) and well aligned (d) with the NV centers family. The misalignment induces a loss of contrast and increases the energy gap at the level anti-crossing, which is of 50 MHz in (c) and less than 5 MHz in (d). The additional ODMR lines are due to the harmonics of the RF generator (fig. 4e of the main text).

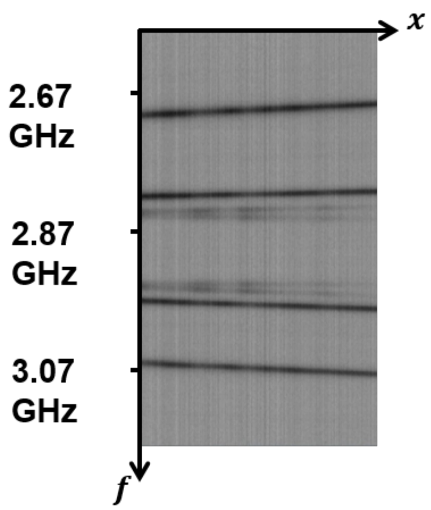

(a) *Mis-aligned static magnetic field*

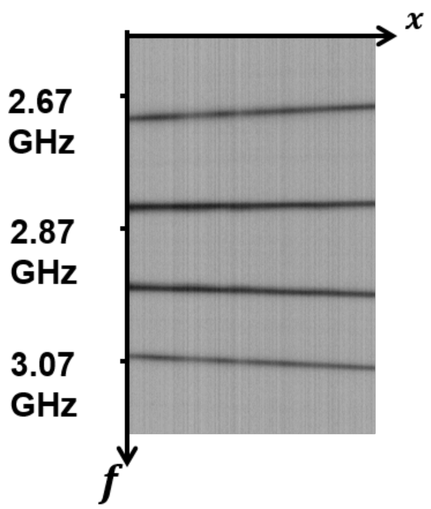

(b) *Aligned static magnetic field*

Supplementary figure 4: Far field alignment procedure. (a) The static magnetic field is not completely aligned with respect to one NV center family. The four NV families undergo a different Zeeman shift and eight ODMR lines, two for each NV center family, are visible. (b) The static magnetic field is aligned to one NV centers family. The three misaligned families undergo the same Zeeman shift and therefore only four ODMR lines are visible, two for the aligned family and two for the three misaligned families. The ODMR lines associated to the well-aligned family are those which undergo the higher Zeeman shift.

from the one which is exited [6]. For this reason, one can discuss the polarization of the emitted PL without considering the polarization of the laser pump[1]. In our experimental configuration the two NV centers families laying in the [110] plane are perpendicular to the optical axis of the imaging system (here defined as z). We consider one of this two families and define its NV center axis as the x axis. $\vec{\mathbf{d_1}}$ and $\vec{\mathbf{d_2}}$ are the two electric dipoles associated to the NV center and they lay in the yz plane. According to eq. (5), the PL intensity (I) emitted by the NV centers along the optical axis z can be written as the sum of the PL intensity emitted by the two dipoles (respectively I_1,I_2):

$$I = I_1 + I_2 \propto |(\vec{\mathbf{z}} \times \vec{\mathbf{d_1}}) \times \vec{\mathbf{z}}|^2 + |(\vec{\mathbf{z}} \times \vec{\mathbf{d_2}}) \times \vec{\mathbf{z}}|^2 \tag{6}$$

Considering the first dipole, but the discussion is identical for the second, the cross product $\vec{\mathbf{z}} \times \vec{\mathbf{d_1}}$ is a vector along the x axis and thus the cross product $(\vec{\mathbf{z}} \times \vec{\mathbf{d_1}}) \times \vec{\mathbf{z}}$ is a vector along the y axis. As a consequence, assuming that only the light propagating along the optical axis is detected by the camera, only the field polarized along y contributes to the PL. Therefore, using a polarizer in our imaging system, it is possible to completely suppress the PL emitted by one of the two NV families laying in the [110] plane. This is not the case for the two families that do not lay in the [110] plane, because the PL they emit along the optical axis is not completely polarized in the [110] plane (Supplementary figure 5). These observations illustrate that a polarizer in the PL detection chain is a useful tool to increase the ODMR contrast of some NV center families. The ODMR contrast of an ensemble of NV centers reads as:

$$C_i = \frac{\mathrm{PL}_i(\mathrm{RF}_{\mathrm{OFF}}) - \mathrm{PL}_i(\mathrm{RF}_{\mathrm{ON})}}{\sum_{j=1}^{4} \mathrm{PL}_j(\mathrm{RF}_{\mathrm{OFF}})} \tag{7}$$

where the subscripts i and j identify the NV center family and $PL_i(RF_{ON})$ and $PL_i(RF_{OFF})$ are respectively the PL emitted by the NV center family i when the RF field is on resonance and out of resonance with the NV centers transition. The ODMR contrast depends not only on the ratio of the PL emission rate when the NV center is on resonance and out of resonance, as it is for a single NV center, but also on the PL of the other families. Therefore, properly placing the polarizer, it is possible to reduce the PL of some families to increase the contrast of some others.

4 Supplementary Note 4. The effect of the laser power on the CW-ODMR spectrum

The effects of the laser power on both the ODMR contrast and the ODRM linewidth have been investigated by [8, 9]. According to the model developed by [9], the ODMR linewidth $\Delta\nu$ and the ODMR contrast C read as:

$$\Delta\nu = \frac{\Gamma_c^\infty}{2\pi} \sqrt{\left(\frac{s}{s+1}\right)^2 + \frac{\Omega_{\mathrm{R}}^2}{\Gamma_p^\infty \Gamma_p^\infty}} \tag{8}$$

$$C = \Theta \frac{\Omega_{\mathrm{R}}^2}{\Omega_{\mathrm{R}}^2 + \Gamma_{\mathrm{p}}^\infty \Gamma_{\mathrm{c}}^\infty \left(\frac{s}{s+1}\right)^2} \tag{9}$$

[1]Since the interaction between the laser and the NV center is mediated by the same two dipoles, also the NV center pumping process depends on the polarization of the laser light.

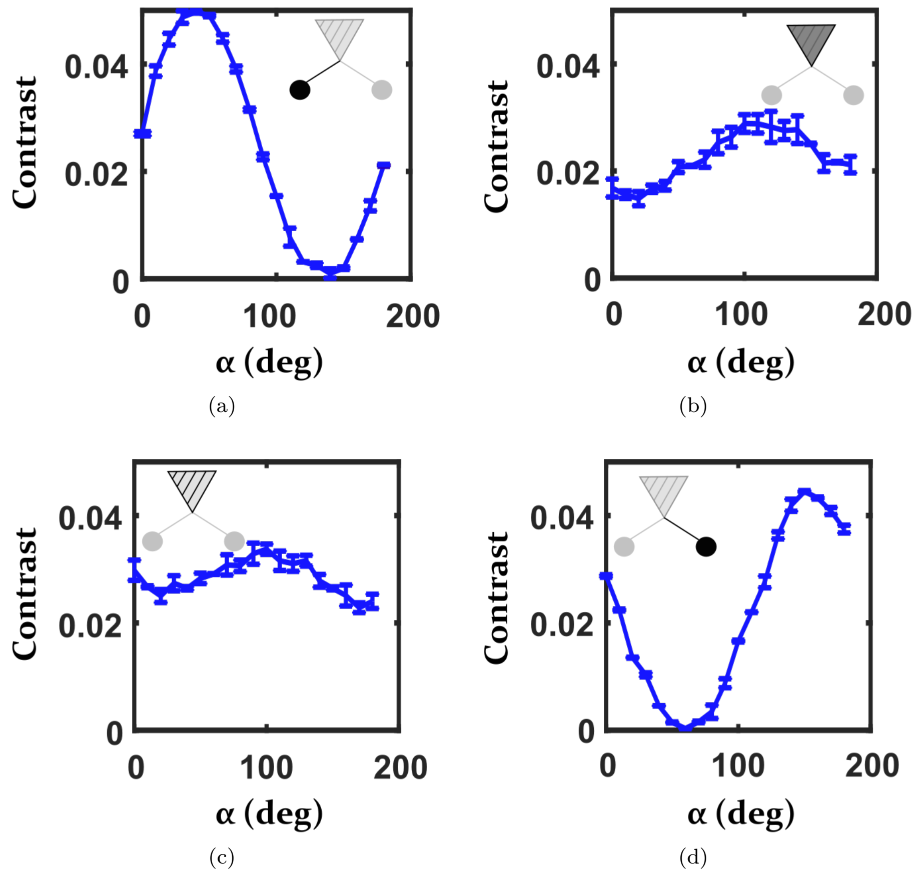

Supplementary figure 5: Contrast variation of the four NV center families tuning the polarizer angle. (a-d) are the contrast of the two NV center families laying in the [110] plane. (b-c) are the contrast of the two NV center families which do not lay in the [110] plane. The orientation of the NV centers family with respect to the [110] plane from which the PL is collected is reported in the wedge-dash diagrams on the top of each plot. Solid lines represent in-plane bonds; Dashed lines represent bonds pointing outside the plane, toward the viewer; wedge-shaped lines represent bonds pointing inside the plane. The error bars correspond to the absolute error between the contrast of the $|0\rangle \rightarrow |{+1}\rangle$ and the $|0\rangle \rightarrow |{-1}\rangle$ transitions of the NV family under analysis.

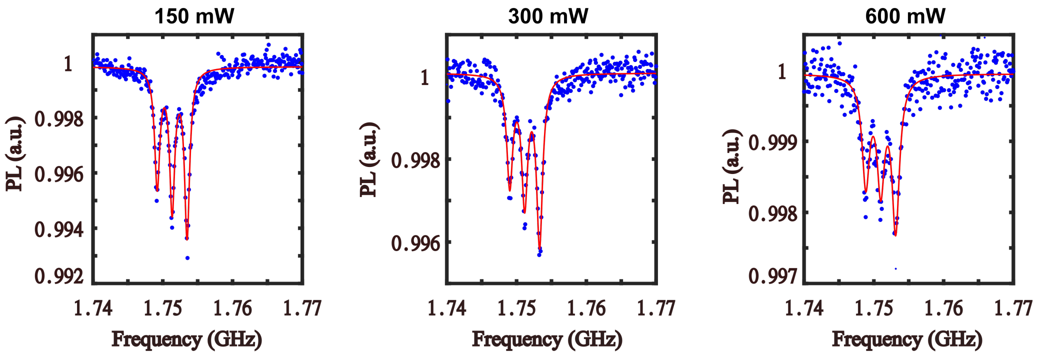

(a) (b) (c)

Supplementary figure 6: Optical power broadening. Comparison between the spectra acquired under different laser powers, reported as the title of each plot. The RF power is set to a nominal value of -5 dBm in order to suppress the RF power broadening. From (a) to (c) the FWHM and the contrast are respectively: FWHM = (0.90 ± 0.04 MHz, 1.04 ± 0.05 MHz and 1.3 ± 0.1 MHz); Contrast=(0.0060 ± 0.0003, 0.004 ± 0.0002, 0.0021 ± 0.0002)

where $\Gamma_c^\infty = 8 \times 10^7$ s^{-1} accounts for the NV center spin decoherence induced by the optical pumping, $\Gamma_p^\infty = 5 \times 10^6$ s^{-1} is the pumping rate when the NV center photoluminescence saturates, Ω_R is the Rabi frequency and it is proportional to the amplitude of the RF driving field and so to the square root of the RF power, Θ is a parameter that accounts for the spin-dependent photoluminescence rate of the NV center and $s = P/P_{sat}$ is the ratio between the laser power and the laser power at the saturation. At low MW power, eq. (8) clearly shows a power broadening effect induced by the laser, while eq. (9) predicts that the contrast decreases when the laser power is increased. These effects are well summarized in Supplementary figure 6, where the ODMR spectra obtained for a MW power of -5 dBm under different laser powers are reported.
It is important to underline that the laser power is one of the several parameters that play a role in the performance of the Q-DiSA architecture and, as it is for all the other parameters, it has to be chosen according to the specification required by the application for which the spectrum analyser is implemented. For instance, in a scenario which requires a high frequency resolution for low power RF signal, the laser power should be set sufficiently low to not compromise the ODMR linewidth and the ODMR contrast. On the contrary, at high RF power, it is advantageous to work with high laser power in order to increase the time resolution of the system and avoid the saturation of the contrast (which affects the dynamic range).
An equivalent approach consists in optimizing the laser power for a given RF power range and implementing an amplification chain which brings the RF signal in that range.

5 Supplementary Note 5. The effect of the magnetic field gradient on the ODMR linewidth and the ODRM Contrast

The magnetic field gradient at the pixel scale induces NV centers imaged by the same pixel to undergo a different Zeeman shift and therefore to be resonant at different frequencies for the same transition (Supplementary figure 7). If the resonance frequency distribution at the pixel scale is small compared to the intrinsic linewidth of the NV center (fig 4a of the main text), the effect of the magnetic field gradient is negligible and all the NV centers imaged by the

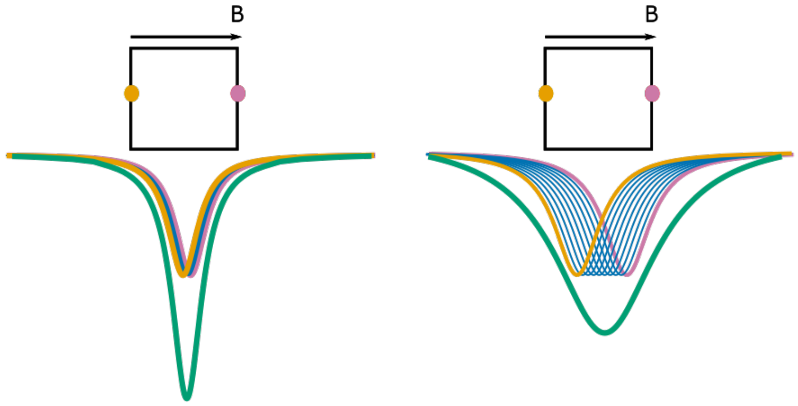

(a) (b)

Supplementary figure 7: Scheme of the effect of the magnetic field gradient per pixel. (a) small magnetic field gradient. (b) strong magnetic field gradient. Assuming the black square to be a pixel, in orange and pink are reported the ODMR spectrum of the NV centers located at two opposite side of the pixel. In blue the ODMR spectrum of the NV centers located inside the pixel. In bluish green the global ODMR spectrum of the pixel, that is the sum of the ODMR spectra of all the NV centers inside the pixel.

same pixel are approximately resonant at the same central frequency (Supplementary figure 7a). On the contrary, if the resonance frequency distribution is comparable or larger than the NV center intrinsic linewidth (fig 4b of the main text), NV centers imaged by the same pixel do not resonate at the same frequency and therefore the ODMR line results broaden and less contrasted (Supplementary figure 7b).
The effect of the static magnetic field gradient can be modelled assuming a linear magnetic field at the pixel scale. The ODMR spectrum $S(\nu)$ associated to a single pixel can then be written as:

$$S(\nu) \propto \int_{\nu_1}^{\nu_2} P(\nu_0) L(\nu - \nu_0)\, d\nu_0 \tag{10}$$

The integration interval $[\nu_1, \nu_2]$ corresponds to the frequency range of the resonance frequencies of the NV centers imaged by the pixel (orange and pink spectra in Supplementary figure 7); $P(\nu_0)$ is the probability distribution of the NV center resonance frequency in the pixel; $L(\nu - \nu_0)$ is the spectrum of a single NV center whose resonance frequency is ν_0. Considering the NV center spectrum as a Lorentzian with a full width at half maximum equals to a, its expression in Eq.10 becomes:

$$L_0(\nu - \nu_0) = \frac{(\frac{a}{2})^2}{(\frac{a}{2})^2 + (\nu - \nu_0)^2} \tag{11}$$

Under the reasonable assumption of a linear magnetic field, the probability distribution $P(\nu_0)$ is uniform in the integration range and therefore it results:

$$S_0(\nu) \propto \int_{\nu_1}^{\nu_2} L_0(\nu-\nu_0)\, d\nu_0 = \int_{\nu_1}^{\nu_2} \frac{(\frac{a}{2})^2}{(\frac{a}{2})^2 + (\nu-\nu_0)^2}\, d\nu_0$$
$$= \frac{a}{2}\left(\arctan\left(\frac{\nu-\nu_2}{a}\right) - \arctan\left(\frac{\nu-\nu_1}{a}\right)\right) \tag{12}$$

Taking into account the hyperfine interaction, the NV center spectrum is described by three Lorentzians frequency shifted by $\nu_{hyp} = 2.14$ MHz. Therefore its expression in Eq.10 becomes:

$$L_{\text{hyp}}(\nu-\nu_0) = \frac{(\frac{a}{2})^2}{(\frac{a}{2})^2 + (\nu-\nu_0+\nu_{\text{hyp}})^2} + \frac{(\frac{a}{2})^2}{(\frac{a}{2})^2 + (\nu-\nu_0)^2} + \frac{(\frac{a}{2})^2}{(\frac{a}{2})^2 + (\nu-\nu_0-\nu_{\text{hyp}})^2} \tag{13}$$

and the single pixel spectrum can be written as:

$$\begin{aligned} S_{\text{hyp}}(\nu) \propto\ & \frac{a}{2}\left(\arctan\left(\frac{\nu-\nu_2+\nu_{\text{hyp}}}{a}\right) - \arctan\left(\frac{\nu-\nu_1+\nu_{\text{hyp}}}{a}\right)\right) \\ & + \frac{a}{2}\left(\arctan\left(\frac{\nu-\nu_2}{a}\right) - \arctan\left(\frac{\nu-\nu_1}{a}\right)\right) \\ & + \frac{a}{2}\left(\arctan\left(\frac{\nu-\nu_2-\nu_{\text{hyp}}}{a}\right) - \arctan\left(\frac{\nu-\nu_1-\nu_{\text{hyp}}}{a}\right)\right) \end{aligned} \tag{14}$$

As we can observe, under a strong magnetic field gradient the ODMR line has not anymore a Lorentzian shape.
A comparison between ODMR spectra under different magnetic field gradient regime is reported in Supplementary figure 8 and Supplementary figure 9.
The theoretical simulation (Supplementary figure 8) is realized considering NV centers with a central frequency uniformly distributed in a frequency range $[-\Delta\nu/2 + \Delta\nu/2]$, where $\Delta\nu = \gamma\Delta B$, γ=28 GHz·T^{-1} is the NV center gyromagnetic ratio and ΔB is the variation of the static magnetic field at the single pixel scale. Thus eq. (14) is solved for different values of ΔB (from 0.01 MHz·pixel^{-1} to 11 MHz·pixel^{-1}) considering as parameters $\nu_2 = -\nu_1 = \Delta\nu/2$ and $a = 1$ MHz, which is the FWHM of the experimental ODMR spectrum obtained at 5 dBm under a magnetic field gradient of 20 kHz·µm^{-1} and a magnetic field of 10 mT (fig 4a of the main text).
To experimentally measure the effect of the magnetic field gradient on the ODMR spectra, we sum the ODMR spectra obtained for adjacent pixels. This summing procedure is equivalent to increase the magnetic field gradient per pixel but instead of increasing the magnetic field gradient we increase the size of the pixel. In this way, by considering only a single experimental measurement, it is possible to study the behaviour of the ODMR spectra under different magnetic field gradient per pixel. In particular we considered the measurement realized when a magnetic field gradient of 20 kHz·µm^{-1} and a magnetic field of 10 mT are applied to the NV centers (fig. 4a of the main text) so that, summing over the entire area of the AOI, along the laser beam direction, we can investigate a magnetic field gradient per pixel from 13 kHz·pixel^{-1} to 10 MHz·pixel^{-1} (summing over $\approx$ 800 pixels).
The results are reported in Supplementary figure 9. The shape of the spectrum is in good agreement with the theoretical model and we can notice that, increasing the gradient, the three peaks of the hyperfine structure are not visible anymore. The contrast and the FWHM of the

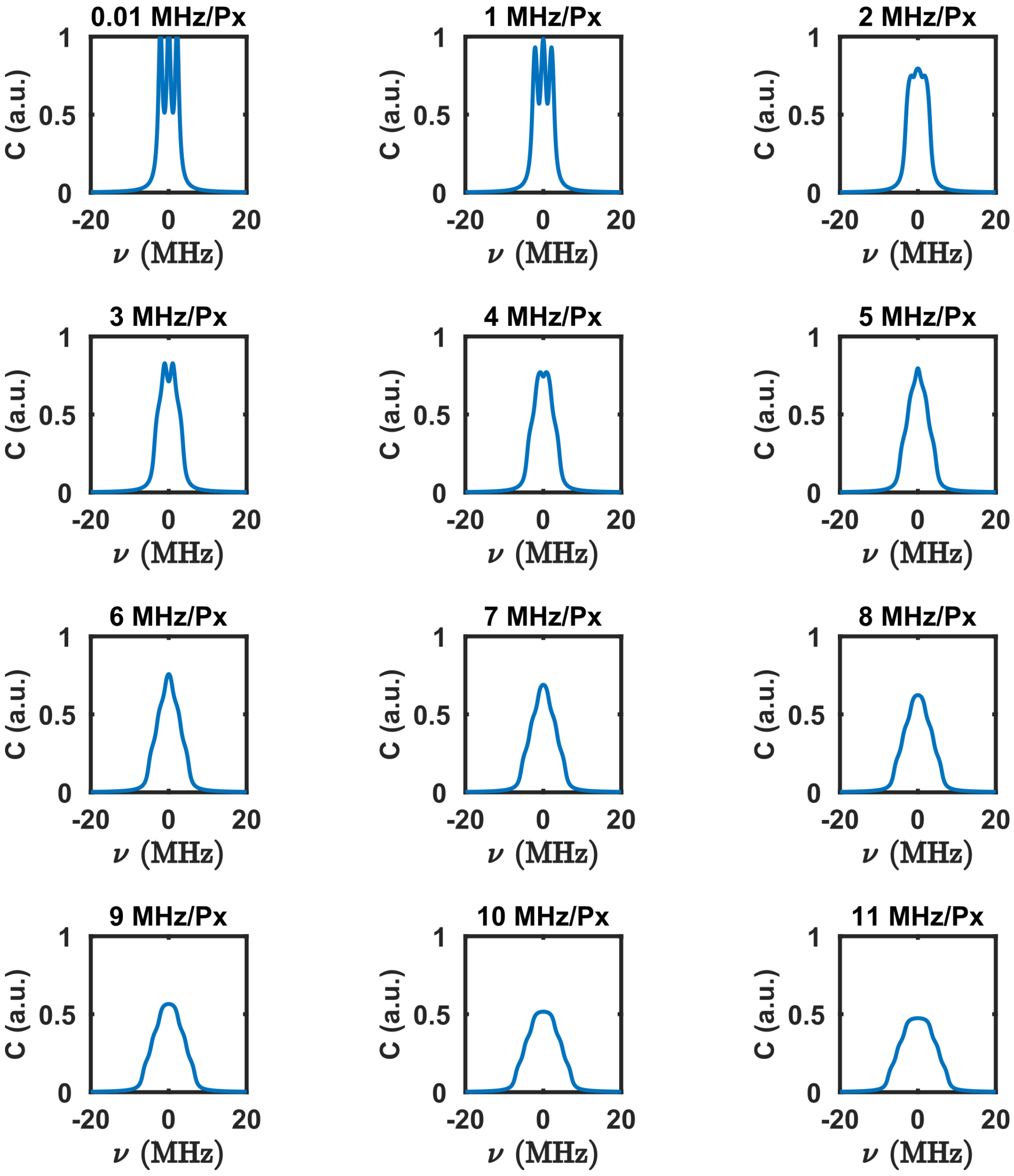

Supplementary figure 8: Simulation of the linewidth broadening caused by the static magnetic field gradient. In the simulation the detuning is considered with respect to the central frequency of the central hyperfine peak. The magnetic field gradient considered for each plot is reported in the title of each plot.

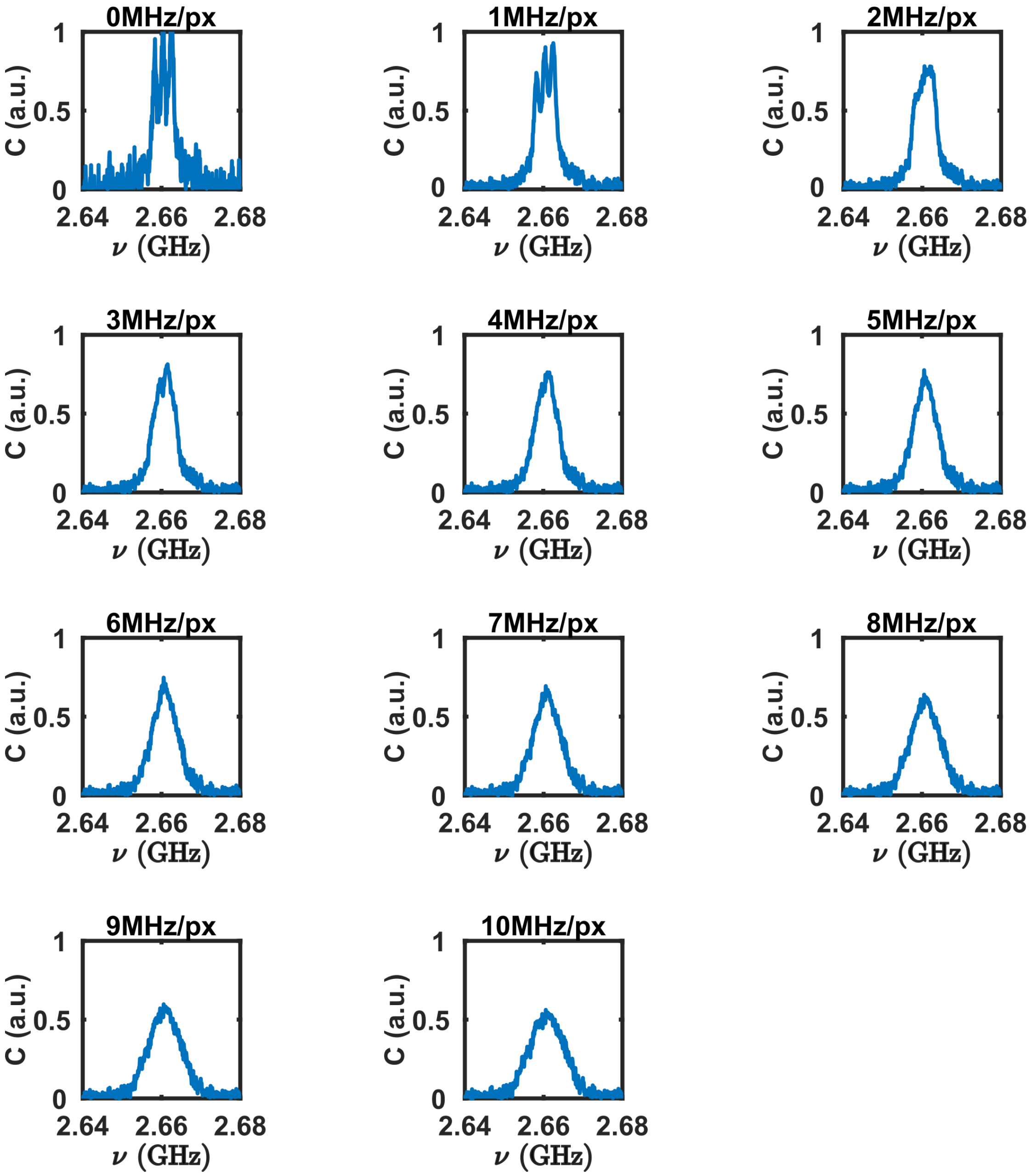

Supplementary figure 9: Experimental measurement of the linewidth broadening caused by the static magnetic field gradient. In order to increase the magnetic field gradient per pixel we summed the spectra of adjacent pixels along the beam propagation axis after having normalized their contrast.

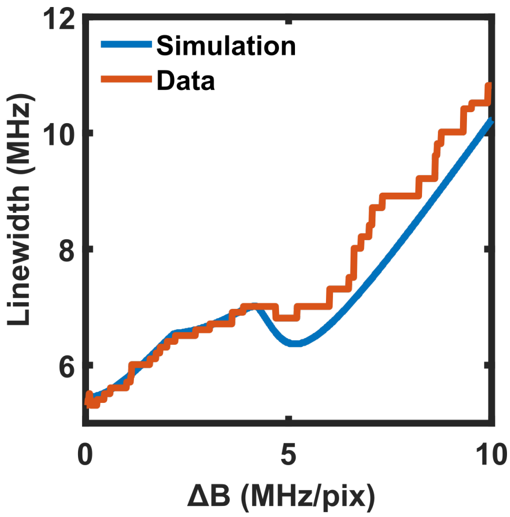

(a)

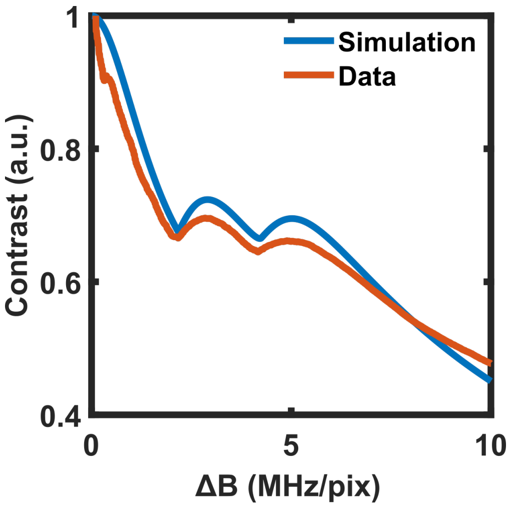

(b)

Supplementary figure 10: Linewidth (a) and contrast (b) evolution under different static magnetic field gradients. The contrast is evaluated as the maximum value of the spectrum while the FWHM is evaluated as the frequency range for which the contrast is higher than half of the maximum value. The simulation and the experimental results are compared showing a good agreement.

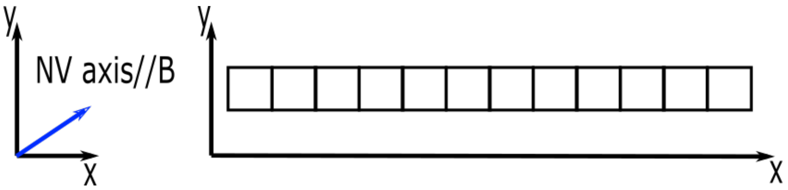

Supplementary figure 11: The NV center axis and the magnetic field (blue arrow) form an angle of $\approx 35°$ with respect to the beam propagation axis (x). Integrating over several pixels along the beam propagation axis (x) we define the magnetic field gradient of the measurement as: $\Delta B = dB/dx \times \ l_{\text{pixel}} \times \ N_{\text{pixel}}$ where dB/dx is the magnetic field gradient along the beam propagation axis, l_{pixel} is the side of the area imaged by a single pixel and N_{pixel} is the number of pixels summed. It is important to remark that the definition of ΔB does not take into account the magnetic field gradient along the two directions perpendicular to the beam propagation axis (y and z). Therefore using ΔB as magnetic field gradient of the measurement results in an underestimation of the real magnetic field gradient.

peaks versus the magnetic field gradient are reported in Supplementary figure 10 and show a good agreement between the experimental results and the simulation. Concerning the FWHM (Supplementary figure 10a), the data are in agreement with the simulation for small magnetic field gradient. The slight disagreement at high magnetic field gradient is likely due to the way we integrate the ODMR spectra over a large number of pixels. In fact, in order to preserve the same pumping parameter, the integration is done along the beam propagation axis, which is not the magnet magnetization axis. Therefore, the magnetic field gradient reported in Supplementary figure 10 is the magnetic field gradient along the beam propagation axis and it does not take into account the magnetic field gradient in the two other directions perpendicular to the beam propagation axis (Supplementary figure 11). On the contrary, the simulation assumes a magnetic field gradient along the magnet magnetization axis, that is the NV center axis. As a consequence, the experimental results are evaluated underestimating the magnetic field gradient and that can explain the small disagreement with the simulation.

The simulation has been realized using a NV center intrinsic linewidth equal to 1 MHz, which corresponds to the ODMR linewidth measured under a RF power of 5 dBm, a static magnetic field of 10 mT and a static magnetic field gradient of 20 kHz$\cdot$pixel^{-1} (fig 4a of the main text). Therefore we can use the simulation to estimate the ODMR linewidth expected for a RF power of 5 dBm under a static magnetic field of 195 mT and a magnetic field gradient of 1.5 MHz$\cdot$pixel^{-1} (fig 4b of the main text): it should be approximately 6 MHz at 5 dBm. The experimental result is (11.5 $\pm$ 1.5) MHz, almost twice the expected value.

The difference between the experimental result and the simulation can be attributed to fluctuations of the magnetic field amplitude during the measurement time. These fluctuations can be caused by both mechanical vibrations, which change the magnet-diamond distance, and temperature fluctuations, which change the magnet remanent magnetization (eq. (2)). In both cases, the effect of these fluctuations is more evident for strong static magnetic field and thus strong magnetic field gradient.

References

[1] David P. Arnold and Naigang Wang. Permanent magnets for mems. *Journal of Microelectromechanical Systems*, 18(6):1255–1266, 2009.

[2] JP Tetienne, L Rondin, P Spinicelli, M Chipaux, T Debuisschert, JF Roch, and V Jacques. Magnetic-field-dependent photodynamics of single NV defects in diamond: an application to qualitative all-optical magnetic imaging. *New Journal of Physics*, 14(10):103033, 2012.

[3] Marcis Auzinsh, Andris Berzins, Dmitry Budker, Laima Busaite, Ruvin Ferber, Florian Gahbauer, Reinis Lazda, Arne Wickenbrock, and Huijie Zheng. Hyperfine level structure in nitrogen-vacancy centers near the ground-state level anticrossing. *Phys. Rev. B*, 100:075204, Aug 2019.

[4] V. Jacques, P. Neumann, J. Beck, M. Markham, D. Twitchen, J. Meijer, F. Kaiser, G. Balasubramanian, F. Jelezko, and J. Wrachtrup. Dynamic polarization of single nuclear spins by optical pumping of nitrogen-vacancy color centers in diamond at room temperature. *Phys. Rev. Lett.*, 102:057403, Feb 2009.

[5] G. Davies, M. F. Hamer, and William Charles Price. Optical studies of the 1.945 ev vibronic band in diamond. *Proceedings of the Royal Society of London. A. Mathematical and Physical Sciences*, 348(1653):285–298, 1976.

[6] RJ Epstein, FM Mendoza, YK Kato, and DD Awschalom. Anisotropic interactions of a single spin and dark-spin spectroscopy in diamond. *Nature physics*, 1(2):94–98, 2005.

[7] Motoichi Ohtsu and Kiyoshi Kobayashi. *Optical Near Fields*. Springer, 2004.

[8] Linbo Shao, Ruishan Liu, Mian Zhang, Anna V. Shneidman, Xavier Audier, Matthew Markham, Harpreet Dhillon, Daniel J. Twitchen, Yun-Feng Xiao, and Marko Lončar. Wide-field optical microscopy of microwave fields using nitrogen-vacancy centers in diamonds. *Advanced Optical Materials*, 4(7):1075–1080, 2016.

[9] A. Dreau, M. Lesik, L. Rondin, P. Spinicelli, O. Arcizet, J.-F. Roch, and V. Jacques. Avoiding power broadening in optically detected magnetic resonance of single NV defects for enhanced dc magnetic field sensitivity. *Phys. Rev. B*, 84:195204, Nov 2011.
